# Cardiac Ischemia Associated With Marijuana Use in an Adolescent

**DOI:** 10.7759/cureus.9661

**Published:** 2020-08-11

**Authors:** Matthew D Schreier, Collette Williams, T. Marsha Ma

**Affiliations:** 1 Internal Medicine-Pediatrics, Ohio State University/Nationwide Children's Hospital, Columbus, USA; 2 Internal Medicine-Pediatrics, Loyola University Medical Center, Maywood, USA; 3 Pediatric Cardiology, Loyola University Medical Center, Chicago, USA

**Keywords:** cardiology, cardiac ischemia, marijuana use, toxicology, adolescent, coronary vasospasm

## Abstract

With the increasing use of marijuana globally, including in pediatric populations, healthcare providers see more adverse health effects associated with this substance. This report details a case presentation of cardiac ischemia in an adolescent male associated with marijuana use. The patient presented with palpitations and chest pain shortly after consuming marijuana. Clinical workup demonstrated anterior ST-segment elevations and troponin elevation with no inflammatory marker elevation or findings of myocarditis on MRI. Extensive drug screening was only positive for marijuana, with a synthetic THC panel negative. These findings resolved after close observation and the patient was discharged in good clinical condition. This case shows a concerning presentation of marijuana-associated cardiac ischemia in an otherwise healthy adolescent, illustrating a potentially severe health concern with an increasingly common substance and demonstrating the need for pediatric centers to have a high index of suspicion for cardiac causes of chest pain when marijuana ingestion is involved even when there are no prior medical or cardiac risk factors.

## Introduction

Marijuana is the most widely-used illicit drug in the United States, with nearly 22.2 million users each month and 38% of high school students reporting marijuana use [1]. Now increasingly championed for its positive health effects, it also possesses an array of adverse effects. While the neuropsychiatric, gastrointestinal, and pulmonary side effects are more well-known, the cardiovascular effects of marijuana still largely have yet to be elucidated, and those that are reported are largely in the adult population [2-5]. Here we present a case report of an otherwise-healthy 17-year-old male presenting with signs of ischemic cardiac disease shortly after ingestion of marijuana.

## Case presentation

A 17-year-old Hispanic male with no prior medical, cardiac, or known substance abuse history and no familial cardiac history presented to an outside hospital approximately 12 hours after feeling a sensation of “heart pounding”, chest pain, and dizziness that began a few hours after work as a furniture mover. The chest pain was localized to his left-central chest and radiated with pain and numbness to his jaw and lower face. The patient was hemodynamically stable with no notable vital sign changes. He was noted to have an elevated qualitative iStat troponin and electrocardiogram (ECG) showing ST-segment elevations in V2 and V3, but no other lab abnormalities including a normal erythrocyte sedimentation rate (ESR), C-reactive protein (CRP), and white blood cell (WBC) count. The patient reported that he had used marijuana via a vaporizer three-to-four hours before the incident. He had no recent illnesses and reported no sick contacts, recent travel, or use of any other substances including cocaine. He was placed on telemetry for the duration of his admission.

**Figure 1:**
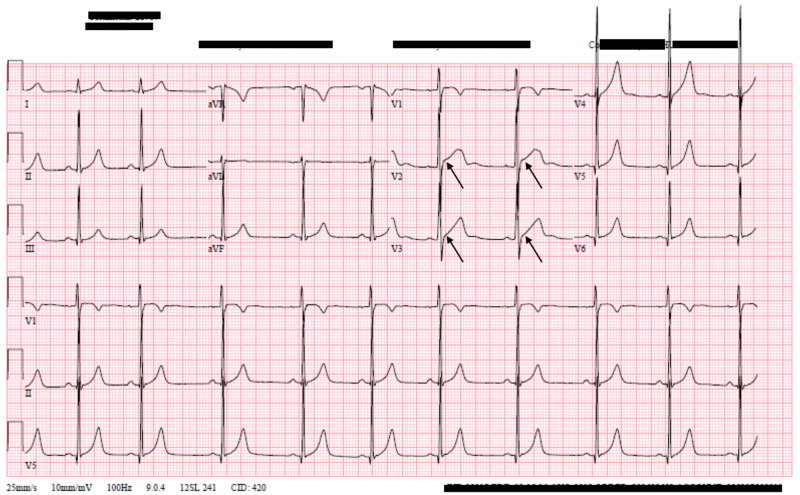
Admission ECG demonstrating ST-elevations in V2 and V3 ECG, electrocardiogram

On admission to our institution, approximately 24 hours after the initiation of symptoms, his troponin I level was elevated to 4.54 ng/mL (normal range 0.00-0.04 ng/mL), his ECG was notable for persistent ST-segment elevation in V2 and V3, CRP and ESR were both within normal limits (0.5 mg/dL and 4 mm, respectively), and his creatine kinase, creatinine, and serum lipids were within normal limits (Figure 1). His urine drug screen was positive for cannabinoids and negative for cocaine and opiates. Screening for synthetic cannabinoids was also negative. An echocardiogram on admission showed no abnormal findings, and a respiratory panel was done with no viruses detected. He had no murmurs or extra sounds on cardiac exam and his physical exam was otherwise unremarkable. The patient at this time reported continued chest pain, but his palpitations and dizziness had resolved.

One day into hospitalization (two days after initiation of symptoms), his troponin I had downtrended to 3.09 ng/mL and no acute events were noted on telemetry. The patient reported improvement in his clinical condition, with a decrease in chest pain and no recurrences of palpitations. Vital signs remained stable throughout admission. A cardiac MRI was performed to rule-out inflammatory or structural heart disease and showed no evidence of inflammation, fibrosis, myocarditis, infarction, or altered perfusion as well as no anomalous origins of coronary arteries.

**Figure 2:**
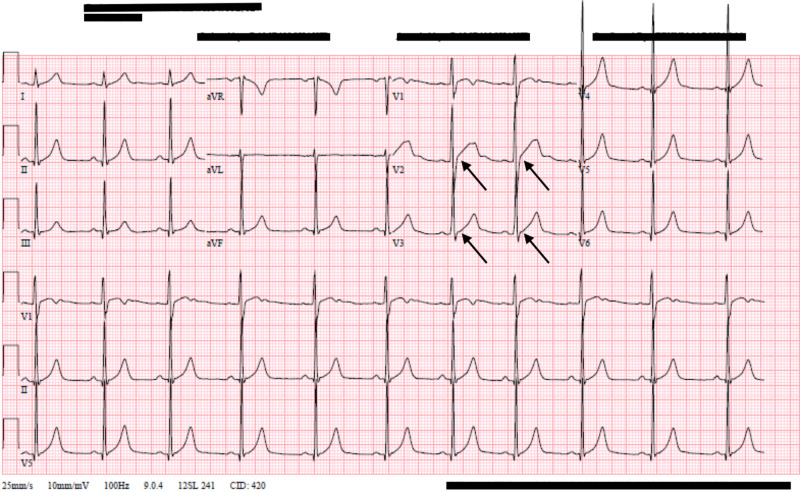
Repeat ECG demonstrating improvement in ST-elevations in V2 and resolution of ST-elevations in V3 ECG, electrocardiogram

By hospital day two, his troponin I had decreased to 2.62 ng/mL, he had no recurrence of chest pain or palpitations, and he was discharged home after a discussion on the importance of avoiding marijuana and with the plan to follow-up as an outpatient (Figure 2).

At one-month outpatient follow-up, the patient reported no recurrence of symptoms and at this time had a normal troponin I level. Additionally, the importance of avoiding marijuana in the future was re-iterated and drug abuse counseling was recommended.

## Discussion

The lack of inflammatory markers in concert with ST-segment elevations and an elevated troponin I level indicate an ischemic etiology of chest pain, and the close proximity of marijuana usage to symptoms suggests a diagnosis of marijuana-induced vasospasm of the coronary arteries.

While the cardiovascular effects of marijuana still have yet to be fully elucidated, it has been shown to have ischemic, arrhythmogenic, and possibly inflammatory effects on the heart. These are largely seen in adults, consistent with the expected higher burden and prevalence of coronary artery disease in the adult population, but these manifestations are being seen in the pediatric and young adult populations as well. Several case reports describe patients meeting criteria for myocardial infarction by ECG and cardiac enzymes after marijuana ingestion in young adulthood with no baseline coronary artery disease on angiography or other substances detected on drug screen [6-8]. Another case series shows similar findings in young adults after the consumption of synthetic marijuana, except with notable thrombus burden on cardiac angiogram [9].

There are also reports of arrhythmogenesis in young patients associated with marijuana consumption including Brugada-like ECG findings in a previously healthy 19-year-old male, and cardiac arrest with ECG-captured ventricular fibrillation and baseline QTc prolongation noted after defibrillation in a previously healthy 16-year-old female [10,11].

A prior report also notes cardiac inflammation after marijuana use presenting as recurrent myopericarditis in a 29-year-old previously healthy male with pericardial rub, CRP and troponin I elevation, and ECG demonstrated ST-segment elevation and PR-segment depression [12].

Case reports in adolescent and pediatric populations are slightly more limited, but have noted a 17-year-old male diagnosed with coronary vasospasm hours after marijuana ingestion with normal coronary angiography in a presentation similar to the patient detailed above, and sudden cardiac arrest in two adolescent males with ECG-demonstrated ventricular fibrillation after ingestion of synthetic cannabinoids [13,14].

Diagnostic workup in these patients commonly includes cardiac enzymes, inflammatory markers, ECG, echocardiogram, and commonly coronary angiography and cardiac MRI depending on the clinical presentation. Management was based on the presenting manifestation, ranging from supportive care to invasive cardiac procedures as indicated.

There are several hypotheses for the pathogenesis of the cardiovascular effects of marijuana. The active ingredient of marijuana, tetrahydrocannabinol (THC), binds to two major receptors in the body, the CB1 which is found in the myocardium and vascular endothelium and likely attenuates the cardiovascular effects, and the CB2 receptor [15]. THC is known to increase sympathetic effects and decrease parasympathetic effects via norepinephrine signaling, having been shown elevate heart rates by 20%-100%, increase cardiac output by 30%, and raise both systolic and diastolic blood pressures [10,15]. It has also been demonstrated to elevate carboxyhemoglobin levels, increasing myocardial oxygen demand and decreasing supply, as well as to decrease time to angina in regular users of the drug [15,16]. There is also data to support that, similar to the well-known effects of cocaine on the coronary arteries, marijuana use can lead to coronary vasospasm in otherwise normal coronary arteries shortly after exposure, generating a vasospastic angina akin to that seen in the patient described in this report [15,16]. Furthermore, there is data to suggest possible effects of THC leading to plaque instability and a procoagulation state in patients with underlying coronary atherosclerosis and thrombosis [9,15].

Overall, there is increasing evidence for a temporal relationship between marijuana and cardiac ischemia, with one study demonstrating a 4.8x risk of acute myocardial infarction in the first hour after marijuana ingestion, and another study showing a hazard ratio of 2.5 for cardiovascular death in those who used marijuana less than weekly and a hazard ratio of 4.2 who used it more than weekly [17,18]. The data in adult patients demonstrate an overall increase in cardiovascular mortality in marijuana users compared to non-users [16]. It is also reported that the use of synthetic cannabinoids is independently associated with cardiovascular risk, and that synthetics overall have increased cardiovascular effects [3,9].

## Conclusions

With the increasing use of and access to marijuana both medicinally and recreationally in children and adolescents as well as an increasing volume of health concerns associated with smoking and vaping marijuana, it is becoming increasingly essential to be aware of the possible clinical manifestations that could lead users to seek medical care. Current data, still largely in the form of case reports, strongly suggests a cardiac toxicity associated with cannabinoids. While some of the cardiac effects of marijuana have been previously borne out, presentations of acute coronary syndrome, coronary vasospasm, and certain life-threatening arrhythmias are still seen largely in the adult population. As coronary artery disease is significantly less prevalent in pediatrics, it is essential to have a high index of suspicion in pediatric centers to ensure these cases aren't overlooked, and this case adds to the body of literature that these presentations are not limited to the population that will be presenting to adult centers. In a pediatric patient presenting with chest pain, palpitations, and dyspnea, it is important to keep coronary artery disease and new-onset arrhythmias on the differential diagnosis, even if there are no personal or familial cardiac risk factors. Marijuana use should be considered in this differential diagnosis. Even if initial testing for marijuana is negative, consideration can be made to test for newer synthetic cannabinoids if there is a reason for suspicion. Furthermore, when providers are counseling pediatric patients about substance use, it is important to pay attention to these often less-known medical complications, as these may prove to have significant effects on morbidity and mortality.
